# Polysubstance use in Canada: prevalence, patterns, and self-reported harms from the 2023 Canadian Substance Use Survey

**DOI:** 10.1186/s12889-026-28095-5

**Published:** 2026-06-23

**Authors:** Ovie Martin Albert, Alexander Arthur

**Affiliations:** 1 VODP Addiction and Mental Health, Centennial Centre for Mental Health and Brain Injury, Alberta Health Services/Recovery Alberta, Ponoka, Canada; 2 Department of Family Medicine, Cumming School of Medicine, University of Calgary, Calgary, Alberta Canada; 3 Department of Family Medicine, University of Saskatchewan, Saskatoon, Saskatchewan Canada; 4 St. Joseph’s Hospital, Estevan, SK Canada

**Keywords:** Polysubstance use, Alcohol co-use, Substance-related harms, Population-based survey, Canada

## Abstract

**Background:**

Polysubstance use contributes substantially to substance-related morbidity and mortality in Canada, yet contemporary population-level estimates of alcohol-involved polysubstance use are limited. We estimated national prevalence patterns and examined associations with self-reported harms using 2023 Canadian Substance Use Survey (CSUS) data.

**Methods:**

We conducted a cross-sectional analysis of the 2023 CSUS public-use microdata file, including Canadians aged ≥ 15 years residing in the provinces. Past-year substance-use patterns were categorized as single-substance use, drug-only polysubstance use (≥ 2 drug classes without alcohol), and drug-plus-alcohol polysubstance use (alcohol plus ≥ 1 drug class). Weighted prevalence estimates were calculated using survey weights and 1,000 bootstrap replicate weights. Survey-weighted logistic regression examined associations with any self-reported harm. A primary model adjusted for sociodemographic confounders; a sensitivity model additionally adjusted for self-rated physical and mental health.

**Results:**

Past-year polysubstance use was reported by 27.2% (95% CI 26.0–28.5) of respondents and was predominantly alcohol-involved (27.0%, 95% CI 25.7–28.3). Drug-only polysubstance use was uncommon (0.3%, 95% CI 0.2–0.4). Common patterns included alcohol plus cannabis (14.4%) and alcohol plus stimulants (11.6%). Compared with single-substance use, drug-plus-alcohol polysubstance use was associated with reporting any harm (adjusted odds ratio [aOR] 4.80; 95% CI 1.02–22.69). Results were robust in sensitivity analyses adjusting for self-rated health (aOR 4.41) and using a stricter concurrent-use definition (aOR 5.72).

**Conclusions:**

Past-year polysubstance use is common in Canada and is largely driven by alcohol co-use. Integration of alcohol into polysubstance prevention, screening, and surveillance frameworks may strengthen public health responses to substance-related harms.

## Introduction

Canada continues to experience substantial morbidity and mortality related to substance use, with opioid- and stimulant-related harms remaining a central public health concern. National surveillance and coroner-based investigations consistently demonstrate that substance-related toxicity deaths rarely involve a single drug in isolation, instead reflecting widespread multi-drug involvement [1–3]. Alcohol, benzodiazepines or other sedatives, and stimulants frequently co-occur with opioids in fatal overdose events across multiple Canadian jurisdictions [1–4]. These findings underscore the growing importance of polysubstance use as a population-level public health issue.

Much of the current evidence on polysubstance-related harms derives from mortality surveillance, toxicology, and administrative data, which are essential for understanding severe outcomes but provide limited insight into population prevalence and non-fatal harms [1–4]. At the same time, the unregulated drug supply in Canada has become increasingly complex and unpredictable, with growing involvement of multiple substance classes, including sedative-type substances [5–7]. While community-based drug-checking and urinalysis programs offer early warning signals regarding changes in supply composition [6, 7], they are not designed to estimate national prevalence or characterize substance-use patterns in the general population. Population-based survey data are therefore critical to contextualize surveillance findings and inform prevention and health promotion strategies.

Polysubstance use is commonly defined as the use of two or more substance classes over a given time period, not necessarily simultaneously [8, 9]. Evidence from cohort and pharmacologic studies indicates that some combinations, particularly opioid-stimulant co-use, are associated with elevated overdose risk and interacting effects [10–12]. Epidemiologic evidence also shows increased overdose risk when opioids are combined with central nervous system depressants such as benzodiazepines/sedatives and alcohol, including in treatment-linked populations [13–15]. Qualitative and mixed-methods syntheses further suggest that polysubstance patterns may be context-dependent, reflecting motivations such as enhancement of desired effects, mitigation of withdrawal symptoms, self-treatment of psychological or physical distress, or substitution when preferred substances are unavailable [8, 16]. This study’s operationalization aligns with established definitions and assessment frameworks that distinguish polysubstance patterns by substance class and context [16]. Alcohol remains especially salient given its high population prevalence and frequent co-use with other substances [17].

Despite extensive evidence linking polysubstance exposure to severe outcomes, much of the existing literature derives from coroner, toxicology, or administrative health data, which are essential for understanding mortality and acute morbidity but provide limited insight into population-level exposure patterns or less severe harms [1–4]. These data sources document the consequences of polysubstance use but do not quantify how widespread such patterns are in the general community, nor how they relate to self-reported health, social, and functional impacts. Population-based survey data are therefore critical for contextualizing mortality surveillance and informing upstream prevention and health promotion strategies.

The Canadian Substance Use Survey (CSUS) offers a recurring, nationally representative platform for characterizing substance-use patterns and associated self-reported harms among Canadians aged ≥ 15 years residing in the provinces [18, 19]. Qualitative evidence from treatment settings further highlights how concurrent stimulant use, including alongside opioid agonist therapy, shapes substance-use patterns and related harms among people who use drugs [20]. By capturing alcohol and drug use across a broad segment of the population, CSUS enables assessment of polysubstance exposure beyond narrowly defined high-risk groups and fatal outcomes, complementing existing surveillance systems.

The CSUS has undergone several iterations since its inception. It evolved from the Canadian Alcohol and Drug Use Monitoring Survey (CADUMS, 2008–2012) through the Canadian Tobacco, Alcohol and Drugs Survey (CTADS, 2013–2017) and the Canadian Alcohol and Drugs Survey (CADS, 2019). In 2023, substantial methodological changes were implemented, including a shift from household sampling to random digit dialling supplemented by probability panel recruitment of youth, and the survey was renamed the Canadian Substance Use Survey. Due to these methodological changes, Health Canada does not endorse direct comparison between CSUS 2023 and prior waves; the present analysis therefore uses CSUS 2023 data as a standalone cross-sectional assessment.

Using CSUS 2023 data, this study aimed to: (i) estimate the prevalence of past-year polysubstance use and key patterns, including alcohol-involved polysubstance use; (ii) describe selected combinations of public health concern; and (iii) examine associations between past-year polysubstance use and self-reported harms in the general population.

## Methods

### Study design and data source

We conducted a cross-sectional secondary analysis of data from the Canadian Substance Use Survey (CSUS) 2023 Public Use Microdata File (PUMF) [21]. CSUS is a nationally representative survey of Canadians aged ≥ 15 years residing in the provinces, designed to monitor alcohol and drug use patterns and associated impacts at the population level. The CSUS sampling frame excludes residents of the territories and selected institutional or non-household populations, as detailed in the survey’s technical documentation [19, 21].

### Study population

All respondents included in the CSUS 2023 PUMF were eligible for descriptive analyses of past-year substance-use patterns. For regression analyses examining associations between substance-use patterns and harms, the analytic sample was restricted to respondents reporting any past-year alcohol and/or drug use, consistent with the attribution of reported harms to substance exposure [2].

### Measures

#### Exposure: past-year substance-use patterns

Past-year substance-use patterns were derived from CSUS self-reported measures of alcohol use and drug use in the preceding 12 months. Respondents were classified into mutually exclusive categories: (1) no past-year alcohol or drug use; (2) single-substance use, subdivided into alcohol-only use (no past-year drug use) and use of a single drug class without alcohol; and (3) polysubstance use. Polysubstance use was further subdivided into drug-only polysubstance use (≥ 2 drug classes without alcohol) and drug-plus-alcohol polysubstance use (alcohol plus ≥ 1 drug class) [3, 4]. Polysubstance exposure was defined over the past 12 months and did not require simultaneous co-ingestion.

Drug classes were operationalized using CSUS past-year indicators for cannabis, opioids, stimulants, and sedatives or tranquilizers, with alcohol treated as a distinct exposure category. Tobacco or nicotine products and prescribed medications used as directed were not included as drug classes in the polysubstance definition [3, 4]. The stimulants category included both illegal stimulants (e.g., cocaine, methamphetamine) and non-medical use of prescription stimulants, consistent with CSUS variable definitions [21].

Alcohol was treated as a distinct exposure category rather than grouped with other drug classes for three reasons. First, its high population prevalence and legal status distinguish it from illicit and prescription drugs in terms of access patterns and policy context. Second, alcohol has established synergistic pharmacological interactions with opioids, benzodiazepines, and stimulants that are clinically distinct from drug-drug interactions among illicit substances. Third, Canadian substance use surveillance and policy frameworks have historically addressed alcohol through separate streams from illicit drug monitoring, making the alcohol-involved versus drug-only distinction directly relevant to public health practice.

Tobacco and nicotine products were excluded from the polysubstance definition for several reasons. The CSUS tobacco module captures past-30-day cigarette and vaping use rather than past-12-month use, creating a temporal mismatch with the other substance class indicators. Additionally, the CSUS harm module does not assess tobacco-attributed harms, and our focus was on alcohol and drug use patterns and their association with harms as captured in that module. While nicotine co-use is an important component of polysubstance patterns in some populations [9], including tobacco would have substantially inflated polysubstance prevalence and shifted the analytic focus away from the alcohol-drug combinations of primary public health concern in the context of the ongoing overdose crisis.

#### Outcome: any self-reported harm

The primary outcome was any self-reported harm attributed to alcohol or drug use in the past 12 months (binary). Harm was defined as endorsement of at least one adverse consequence across physical health, mental or emotional well-being, social or relationship functioning, financial impact, or work- or school-related problems, as captured in the CSUS harm module [21]. Harm questions were administered only to respondents reporting past-year alcohol and/or drug use.

#### Covariates

The primary regression model adjusted for age group, sex at birth, household income, educational attainment, and homelessness experience, representing the available sociodemographic confounder set in the CSUS 2023 Public Use Microdata File. Marital status and employment, which were collected in the survey, were not available in the PUMF due to Statistics Canada disclosure control procedures. A prespecified sensitivity model additionally adjusted for self-rated physical health and self-rated mental health; these variables were reserved for sensitivity analysis given their ambiguous position as potential confounders or mediators of the exposure-harm relationship in cross-sectional data.

### Statistical analysis

All analyses were conducted using R (version 4.5.1) and the *survey* package. Population-representative point estimates incorporated the CSUS person-level sampling weight to account for the complex survey design [21]. Variance estimation was performed using the 1,000 mean bootstrap replicate weights provided with the CSUS 2023 PUMF, applying replicate-weight survey design methods [2, 21]. Because CSUS replicate weights are supplied as mean bootstrap weights, each representing an average of multiple bootstrap samples, variance estimates were adjusted using the recommended scaling factor, as specified in CSUS technical guidance [21].

Weighted prevalences and corresponding 95% confidence intervals were estimated using bootstrap-based standard errors. Associations between past-year polysubstance-use category and self-reported harm were examined using survey-weighted logistic regression models with bootstrap variance estimation. Regression analyses were restricted to respondents reporting any past-year substance use and compared drug-only polysubstance use and drug-plus-alcohol polysubstance use with single-substance use as the reference category. Models adjusted for age group, sex at birth, household income, educational attainment, and homelessness experience in the primary model. Due to small cell sizes and disclosure control requirements in the public-use microdata file, respondents reporting non-binary or other sex were excluded from regression analyses. Analyses were conducted using complete cases for exposure, outcome, and covariates, and statistical significance was assessed using a two-sided *p* < 0.05 threshold.

In addition to the primary polysubstance categorization, we conducted combination-specific analyses examining the association between selected substance co-use combinations and self-reported harm. Candidate combinations were assessed for analytic feasibility based on unweighted cell sizes and disclosure constraints; only combinations with adequate sample sizes and stable estimates were carried forward. As a further sensitivity analysis, we constructed a concurrent-use polysubstance variable from CSUS items that explicitly capture use of multiple substances at the same time or on the same occasion (ALC_Q17, CAN_Q10, ODS_Q05B), corresponding to Health Canada’s official concurrent-use definition.

### Disclosure control

Disclosure control procedures were applied in accordance with CSUS PUMF reporting guidelines. Estimates were suppressed when unweighted cell counts were small, and estimates with high sampling variability were interpreted cautiously, consistent with public-use microdata standards [21].

### Ethics

This study used de-identified, publicly available microdata and did not involve direct contact with participants; institutional research ethics board review was therefore not required.

## Results

### Sample characteristics

The analytic sample included 36,180 respondents, representing a weighted population of 32,757,730 Canadians aged ≥ 15 years residing in the provinces (Table 1). Drug-plus-alcohol polysubstance users comprised the overwhelming majority of polysubstance users (unweighted *n* = 9,622), whereas drug-only polysubstance use was rare (unweighted *n* = 99).

**Table 1:** Sociodemographic and health characteristics by past-year substance-use category among Canadians aged ≥ 15 years, CSUS 2023 (weighted percentages with 95% confidence intervals)

Characteristic	Total	Single substance/no polysubstance	Drug-only polysubstance	Drug-plus-alcohol polysubstance
Unweighted N	36,180	26,459	99	9,622
Weighted population N	32,757,730	23,824,121	97,870	8,835,739
Age group (years)				
15–24	14.1% [13.6–14.6]	13.6% [13.1–14.1]	15.5% [9.6–21.4]*	15.3% [14.4–16.2]
25–34	16.7% [16.1–17.2]	14.8% [14.2–15.4]	16.5% [10.4–22.6]*	21.9% [20.9–22.9]
35–44	16.1% [15.6–16.6]	14.1% [13.5–14.7]	23.5% [16.4–30.6]*	21.3% [20.3–22.3]
45–54	14.7% [14.2–15.2]	15.0% [14.4–15.6]	17.1% [10.9–23.3]*	13.7% [12.9–14.5]
55–64	16.1% [15.5–16.6]	16.3% [15.7–16.9]	19.5% [12.9–26.1]*	15.3% [14.5–16.1]
65+	22.3% [21.7–22.9]	26.1% [25.4–26.8]	7.8% [3.4–12.2]*	12.4% [11.7–13.1]
Sex at birth				
Male	49.5% [48.8–50.2]	48.7% [47.9–49.5]	67.9% [59.6–76.2]	51.4% [50.2–52.6]
Female	50.5% [49.8–51.2]	51.3% [50.5–52.1]	32.1% [23.8–40.4]	48.6% [47.4–49.8]
Household income				
<$20,000	7.2% [6.8–7.6]	6.9% [6.5–7.3]	14.2% [8.4–20.0]*	7.7% [7.1–8.3]
>$150,000	17.5% [16.9–18.1]	16.7% [16.1–17.3]	8.8% [4.2–13.4]*	19.6% [18.6–20.6]
Self-rated physical health				
Excellent/Very good	55.7% [55.0–56.4]	57.8% [57.0–58.6]	34.8% [26.4–43.2]	50.3% [49.1–51.5]
Good	31.8% [31.1–32.5]	31.0% [30.3–31.7]	32.6% [24.4–40.8]	34.0% [32.9–35.1]
Fair/Poor	12.3% [11.8–12.8]	11.0% [10.5–11.5]	30.8% [22.7–38.9]	15.6% [14.7–16.5]
Self-rated mental health				
Excellent/Very good	53.5% [52.8–54.2]	58.1% [57.3–58.9]	27.4% [19.6–35.2]	41.4% [40.2–42.6]
Good	27.9% [27.2–28.6]	26.8% [26.1–27.5]	24.9% [17.5–32.3]*	31.0% [29.9–32.1]
Fair/Poor	18.4% [17.8–19.0]	14.9% [14.3–15.5]	47.7% [39.2–56.2]	27.3% [26.2–28.4]

Values are weighted percentages with 95% confidence intervals. Estimates incorporate CSUS 2023 person-level survey weights; variance estimation used the CSUS 2023 mean bootstrap replicate weights (1,000 replicates). Estimates marked with an asterisk (*) indicate high variability and should be interpreted with caution. Employment and marital status variables were not available in the Public Use Microdata File due to Statistics Canada disclosure control procedures. Household income categories are shown for the lowest and highest brackets; intermediate categories are omitted for brevity

Drug-plus-alcohol polysubstance use was most prevalent among younger and early middle-aged adults, particularly those aged 25–44 years, and declined markedly among adults aged ≥ 65 years (Table 1). Individuals reporting drug-only polysubstance use had a substantially higher prevalence of fair or poor self-rated mental health compared with those reporting single-substance use or no past-year substance use (Table 1).

### Prevalence of substance-use patterns

Overall, 27.2% (95% CI 26.0–28.5) of respondents reported past-year polysubstance use (Table 2). Nearly all polysubstance exposure involved alcohol co-use (27.0%, 95% CI 25.7–28.3), whereas drug-only polysubstance use was uncommon (0.3%, 95% CI 0.2–0.4).

**Table 2:** Weighted prevalence of past-year substance-use patterns and selected polysubstance combinations among Canadians aged ≥ 15 years, CSUS 2023

Pattern/combination	Unweighted *n*	Weighted % (95% CI)
A. Overall substance-use patterns (mutually exclusive)		
No past-year alcohol or drug use	4,237	11.8% [10.8–12.8]
Single substance use (total)	22,222	61.0% [59.6–62.4]
Alcohol only (no past-year drugs)	21,986	60.3% [58.9–61.7]
Single drug class only (no alcohol)	236	0.7% [0.5–0.9]
Polysubstance use (total)	9,721	27.2% [26.0–28.5]
Drug-only polysubstance (≥ 2 drug classes; no alcohol)	99	0.3% [0.2–0.4]
Drug-plus-alcohol polysubstance (alcohol + ≥ 1 drug class)	9,622	27.0% [25.7–28.3]
Alcohol + 1 drug class	4,972	13.8% [12.8–14.8]
Alcohol + ≥ 2 drug classes	4,650	13.2% [12.2–14.2]
B. Selected common and high-risk combinations (not mutually exclusive)		
Alcohol + cannabis	5,272	14.4% [13.4–15.4]
Alcohol + stimulants (including cocaine)	4,008	11.6% [10.7–12.5]
Cannabis + stimulants	2,062	5.9% [5.2–6.6]
Cannabis + other drugs†	2,791	7.7% [6.9–8.5]
Opioids + benzodiazepines/sedatives	5	F (suppressed)
Opioids + stimulants (e.g., methamphetamine, cocaine)	490	1.4% [1.1–1.7]
Opioids + alcohol	1,070	3.2% [2.7–3.7]
Sedatives + alcohol	18	F (suppressed)

Values are weighted percentages with 95% confidence intervals. Estimates incorporate CSUS 2023 person-level survey weights, with variance estimation conducted using the CSUS 2023 mean bootstrap replicate weights (1,000 replicates). Categories in section B are not mutually exclusive. “F” indicates estimates suppressed due to small unweighted cell counts (< 30), consistent with public-use microdata disclosure requirements. Stimulants include both non-medical use of prescription stimulants and illegal stimulants. †Other drugs include psychedelics (LSD, psilocybin, mescaline), dissociatives (PCP, ketamine), inhalants, salvia, kratom, and nitrous oxide

Single-substance use accounted for 61.0% (95% CI 59.6–62.4) of respondents and was driven primarily by alcohol-only use (60.3%, 95% CI 58.9–61.7). Use of a single drug class without alcohol was rare (0.7%, 95% CI 0.5–0.9).

Among respondents reporting alcohol-involved polysubstance use, similar proportions reported alcohol plus one drug class (13.8%, 95% CI 12.8–14.8) and alcohol plus two or more drug classes (13.2%, 95% CI 12.2–14.2) (Table 2).

### Selected combinations of public health interest

Among selected non-mutually exclusive combinations, the most prevalent co-use patterns were alcohol plus cannabis (14.4%, 95% CI 13.4–15.4) and alcohol plus stimulants (11.6%, 95% CI 10.7–12.5) (Table 2). Cannabis plus stimulants (5.9%, 95% CI 5.2–6.6) and cannabis plus other drugs (7.7%, 95% CI 6.9–8.5) were also observed.

Several high-risk combinations, including opioid-sedative co-use, could not be reliably estimated and were suppressed due to small unweighted cell counts (Table 2).

### Age patterning

**Fig. 1:**
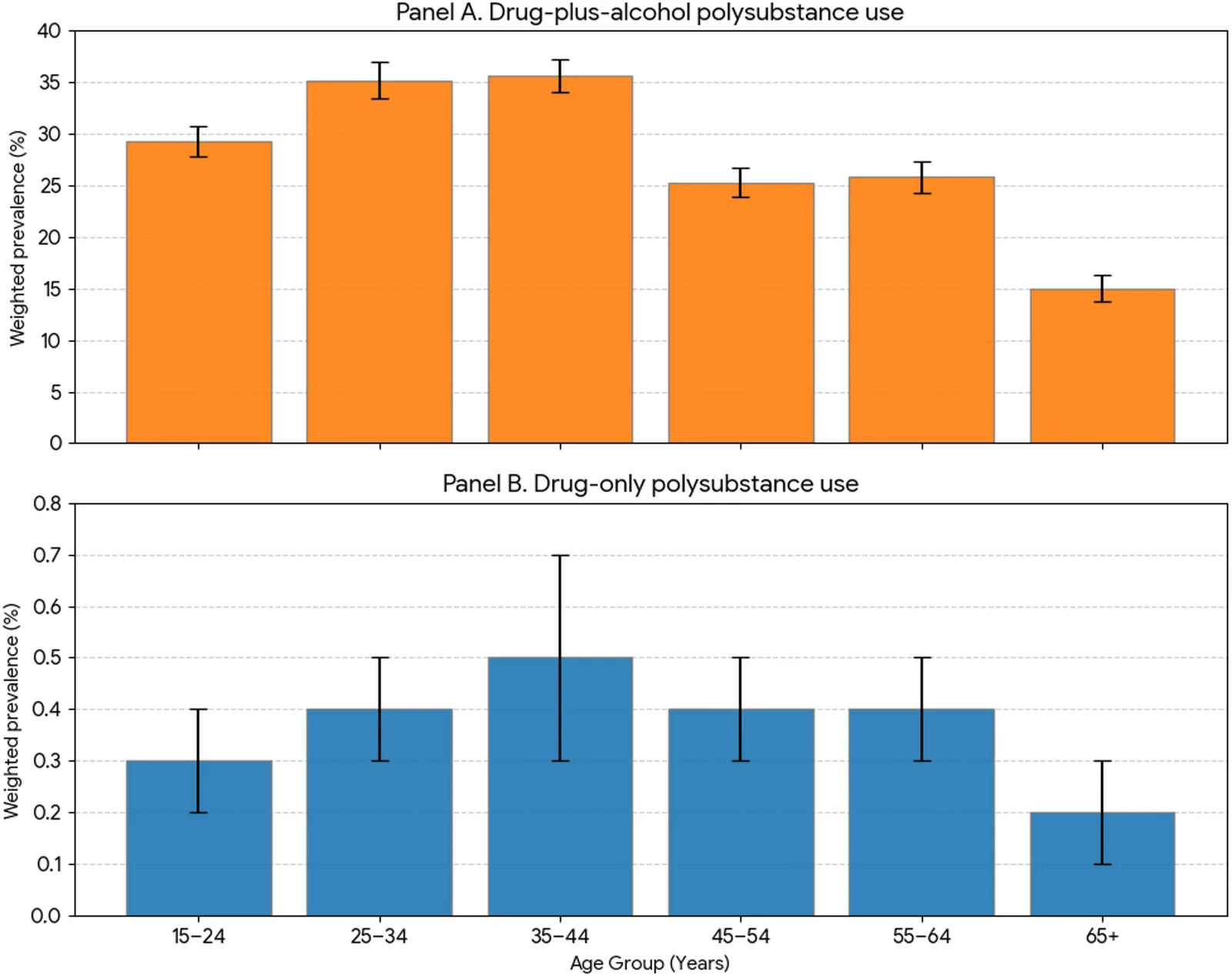
Weighted prevalence of past-year polysubstance use by age group, CSUS 2023. Panel **A** shows the weighted prevalence of drug-plus-alcohol polysubstance use (alcohol plus ≥ 1 drug class). Panel **B** shows the weighted prevalence of drug-only polysubstance use (≥ 2 drug classes without alcohol). Error bars represent 95% confidence intervals estimated using CSUS 2023 mean bootstrap replicate weights (1,000 replicates). Prevalence estimates reflect past-year use and are weighted to the national population

Age-stratified prevalence patterns are shown in Fig. 1. Drug-plus-alcohol polysubstance use was concentrated among younger and middle-aged adults and declined steadily with increasing age, whereas drug-only polysubstance use remained rare across all age groups.

### Association between past-year polysubstance use and self-reported harm

In survey-weighted logistic regression analyses restricted to past-year substance users, drug-plus-alcohol polysubstance use was associated with higher odds of reporting any self-reported harm compared with single-substance use (Table 3). In the primary model adjusting for age, sex, income, education, and homelessness, drug-plus-alcohol polysubstance use was associated with an adjusted odds ratio of 4.80 (95% CI 1.02–22.69; *p* = 0.048). Drug-only polysubstance use showed a large but imprecise point estimate (aOR 17.32, 95% CI 0.14–2,132.78; *p* = 0.245), reflecting the small sample size of this group. In the sensitivity model additionally adjusting for self-rated physical and mental health, the association for drug-plus-alcohol polysubstance use remained significant (aOR 4.41, 95% CI 1.04–18.72; *p* = 0.044), representing an 8.2% attenuation from the primary model.

**Table 3:** Survey-weighted logistic regression examining the association between past-year polysubstance use and any self-reported harm among past-year substance users, CSUS 2023

Exposure (Reference: Single-substance use)	aOR	95% CI	*p*-value
Panel A: Primary model			
Drug-plus-alcohol polysubstance	4.80	1.02–22.69	0.048
Drug-only polysubstance	17.32	0.14–2,132.78	0.245
Panel B: Sensitivity model			
Drug-plus-alcohol polysubstance	4.41	1.04–18.72	0.044
Drug-only polysubstance	15.42	0.20–1,213.39	0.219

Adjusted odds ratios (aORs) and 95% confidence intervals are shown. Reference group is single-substance use. Panel A: primary model adjusted for age group, sex at birth, household income, educational attainment, and homelessness experience (complete cases: 21,840). Panel B: sensitivity model additionally adjusted for self-rated physical health and self-rated mental health (complete cases: 21,807). Models apply CSUS 2023 person-level survey weights with variance estimation using 1,000 mean bootstrap replicate weights

Increasing age was associated with progressively lower odds of reporting harm relative to ages 15–24 years, and females had lower odds of harm than males (Table 3).

### Concurrent-use sensitivity analysis

As a sensitivity analysis, we examined a concurrent-use polysubstance measure constructed from CSUS items that explicitly capture substance co-use at the same time or on the same occasion (ALC_Q17, CAN_Q10, ODS_Q05B). This measure corresponds to Health Canada’s official concurrent-use definition. The prevalence of concurrent polysubstance use was 29.2%, closely matching Health Canada’s published estimate of 28%. The concurrent-use prevalence exceeded the past-year polysubstance prevalence (27.2%) because the concurrent-use items capture alcohol-cannabis same-occasion co-use among respondents who may not have met the drug-class threshold in the primary categorization, which required use of at least one non-alcohol drug class. In survey-weighted logistic regression adjusting for the same sociodemographic covariates as the primary model, concurrent polysubstance use was associated with self-reported harm (aOR 5.72, 95% CI 0.98–33.48, *p* = 0.053; complete cases: 21,872). The point estimate was slightly higher than the primary model (4.80), suggesting that the observed association is unlikely to be solely an artifact of the broader 12-month reference period, although the estimate was imprecise.

## Discussion

### Principal findings

In this national, population-based analysis of CSUS 2023, past-year polysubstance use was common among Canadians aged ≥ 15 years and was overwhelmingly characterized by alcohol co-use rather than drug-only combinations. Although drug-only polysubstance use was rare at the population level, it showed an elevated but uncertain point estimate for self-reported harm, indicating that relatively uncommon exposure profiles may carry disproportionate risk. Together, these findings position polysubstance exposure as a broad public health concern extending beyond narrowly defined high-risk subpopulations.

A central and policy-relevant finding is the predominance of alcohol-involved polysubstance use. While public health responses have largely focused on illicit drug combinations, alcohol involvement has been consistently documented in substance-related toxicity deaths across Canada. National and provincial surveillance, coroner-based analyses, and mortality reviews demonstrate frequent co-occurrence of alcohol with opioids, benzodiazepines, and stimulants in fatal toxicity events [1–4]. The present findings extend this evidence by showing that alcohol-involved polysubstance exposure is also the dominant pattern at the population level, reinforcing the need to integrate alcohol explicitly into polysubstance risk frameworks.

Drug-plus-alcohol polysubstance use remained associated with self-reported harm after adjustment for sociodemographic confounders. Drug-only polysubstance use had an elevated point estimate, but the association could not be estimated precisely due to the small number of respondents in this category (*n* = 99); the confidence interval was extremely wide and included the null value. While the direction of this estimate is consistent with elevated risk, the finding should be interpreted with caution.

### Combination-specific findings

Combination-specific analyses suggested heterogeneity in the association between different substance co-use patterns and self-reported harm (Table 4), though estimates for less common combinations were imprecise and should be interpreted cautiously. The wide confidence intervals observed in several models reflect sparse cell counts and should be interpreted as indicative of direction rather than precise magnitude of association. Alcohol combined with stimulants (aOR 22.27, 95% CI 3.65–135.83) and cannabis combined with stimulants (aOR 22.95, 95% CI 1.79–293.80) showed the largest point estimates, consistent with pharmacological evidence of synergistic cardiovascular and neurotoxic effects, though the wide confidence intervals reflect limited sample sizes for these combinations. Alcohol combined with opioids was also significantly associated with harm (aOR 14.99, 95% CI 1.15–195.15), but the confidence interval spanned more than two orders of magnitude, precluding precise quantification of the effect. Alcohol combined with cannabis, the most prevalent combination, showed a more modest and non-significant association (aOR 4.57, 95% CI 0.87–24.00, *p* = 0.072). Opioids combined with stimulants had a large but extremely unstable point estimate (aOR 61.08, 95% CI 0.19–19,278.81, *p* = 0.161) that cannot be meaningfully interpreted. Opioid-sedative co-use could not be estimated at all due to sparse data. These patterns are broadly consistent with known pharmacological risk profiles but require confirmation in larger datasets or through linkage to administrative health records.

**Table 4:** Survey-weighted logistic regression examining associations between selected substance co-use combinations and any self-reported harm, CSUS 2023

Combination	aOR	95% CI	*p*-value
Alcohol + Cannabis	4.57	0.87–24.00	0.072
Alcohol + Stimulants	22.27	3.65–135.83	< 0.001
Alcohol + Opioids	14.99	1.15–195.15	0.039
Cannabis + Stimulants	22.95	1.79–293.80	0.016
Opioids + Stimulants	61.08	0.19–19,278.81	0.161

Reference group is single-substance users. All models adjusted for age group, sex at birth, household income, educational attainment, and homelessness experience. Each combination was modelled separately against single-substance users. Opioids + sedatives was excluded due to sparse data. Models apply CSUS 2023 person-level survey weights with variance estimation using 1,000 mean bootstrap replicate weights

### Comparison with existing evidence

The association between polysubstance use and elevated risk of acute toxicity and overdose is well established. Cohort and population-based studies consistently show increased overdose risk associated with concurrent opioid-stimulant use and opioid use combined with sedatives or benzodiazepines [10–14]. Pharmacologic evidence further demonstrates synergistic effects of these combinations on respiratory depression, cardiovascular strain, and neurotoxicity, resulting in risk that exceeds additive effects [11, 12].

Beyond pharmacologic mechanisms, qualitative and mixed-methods studies suggest that polysubstance use reflects adaptive behaviours shaped by individual needs and structural conditions. Motivations including enhancement of desired effects, mitigation of withdrawal, self-treatment of mental health symptoms, and substitution in response to drug supply changes have been described across diverse populations [10, 16–20, 22]. Despite this behavioural complexity, the downstream health consequences of polysubstance exposure are well documented [23]. Clinical studies link stimulant co-use to adverse cardiovascular outcomes, including heart failure associated with cocaine [24] and methamphetamine [25], while population-based analyses demonstrate elevated overdose risk associated with concurrent use of Z-drugs and opioids [26]. Recent surveillance data further indicate that polysubstance exposure contributes to accidental acute toxicity deaths among youth [27]. These dynamics are particularly salient in the context of an increasingly volatile unregulated drug supply, where contamination and variability in potency may further amplify risk [28–30].

Much of the existing evidence on polysubstance-related harms derives from coroner, toxicology, and administrative health datasets, which are indispensable for understanding mortality and severe morbidity but provide limited insight into exposure prevalence or non-fatal harms [1–4, 30, 31]. Population-based survey data therefore play a critical complementary role by capturing a broader spectrum of substance-use patterns, including individuals who may not appear in acute care or mortality records. The present study contributes to this evidence base by quantifying polysubstance exposure and associated harms in the general population. Ecological approaches that contextualize enforcement and administrative indicators alongside survey-based prevalence estimates face analogous challenges in reconciling data sources with different coverage and ascertainment properties, as has been demonstrated in other national settings [32].

### Implications for public health policy and practice

The predominance of alcohol-involved polysubstance use has important implications for public health policy and practice. Screening and brief intervention strategies in primary care, emergency departments, and community settings should explicitly assess alcohol co-use alongside other substances, rather than treating alcohol as ancillary to illicit drug use. Evidence from treatment and harm-reduction settings indicates that alcohol use can meaningfully modify risk among people using opioids and other drugs, including those receiving opioid agonist treatment [15]. These findings are particularly relevant for provincial public health agencies and population-level prevention programs, where alcohol and drug use are often addressed through separate policy and surveillance streams.

Prevention and harm-reduction strategies should therefore continue to emphasize avoidance of high-risk combinations, particularly opioid-sedative and opioid-stimulant co-use, while also addressing alcohol’s role in amplifying overdose risk [1, 10–14] Interventions such as naloxone distribution, safer-use education, and tailored counselling may be more effective when framed around polysubstance risk rather than single-substance use.

Although drug-only polysubstance use was uncommon at the population level, the elevated but imprecise point estimate is consistent with this group carrying disproportionate risk, though the small sample size (*n* = 99) precludes firm conclusions. If confirmed in larger samples, this would suggest the presence of a small but high-risk subgroup that may benefit from targeted prevention and clinical outreach strategies. In addition, the observed age distribution indicates that polysubstance-related interventions should not be limited to adolescents or young adults; working-age adults account for a substantial proportion of population-level polysubstance exposure and should be explicitly included in prevention and harm-reduction efforts.

### Study limitations

Several limitations should be considered when interpreting these findings. First, the analysis is cross-sectional; therefore, observed associations cannot establish temporality or causality between polysubstance use and harms. Second, CSUS measures are self-reported and may be subject to recall error and social desirability bias, particularly for stigmatized substances. Third, polysubstance exposure was defined over the past 12 months and did not require simultaneous co-ingestion; as a result, some observed combinations may reflect sequential rather than concurrent use, and associated risk may be over- or under-estimated depending on timing and dose. As a sensitivity analysis, we reconstructed polysubstance use using CSUS concurrent-use items that assess same-occasion co-use; the association with harm was consistent under this stricter definition (see above).

Fourth, the harms outcome is self-attributed to substance use and may not fully capture clinically verified harms or health-service utilization. Individuals may not always accurately identify the substances they have consumed; self-reported substance use may diverge from toxicological verification, particularly for opioids and sedatives in the context of an increasingly complex unregulated drug supply [33, 34]. Fifth, geographic granularity and covariate detail are constrained by the public-use microdata structure, and very small cell sizes necessitate suppression or cautious interpretation for rare polysubstance combinations. Although variance estimation appropriately incorporated the full set of CSUS mean bootstrap replicate weights with recommended scaling, estimates for rare exposure patterns remain imprecise due to limited sample sizes.

Tobacco and nicotine products were excluded from the polysubstance definition due to temporal measurement differences and the absence of tobacco-attributed harms in the CSUS harm module. While this exclusion is consistent with the study’s focus on alcohol-drug combinations, it may underestimate overall polysubstance burden, particularly in populations where tobacco-substance co-use is clinically relevant.

Marital status and employment were collected in CSUS 2023 but were not available in the Public Use Microdata File due to Statistics Canada disclosure control procedures. Education and homelessness experience were used as alternative sociodemographic controls.

## Conclusion

In this national analysis of CSUS 2023, past-year polysubstance use was common among Canadians aged ≥ 15 years and was largely driven by alcohol-involved patterns. Drug-plus-alcohol polysubstance use was associated with self-reported harms, and the point estimate for drug-only polysubstance use, while elevated, was imprecise due to sparse data. Combination-specific analyses suggested heterogeneity across substance co-use patterns, though estimates for less common combinations were imprecise and require confirmation in larger samples. These findings highlight the importance of addressing alcohol co-use within broader polysubstance prevention efforts. Integrating alcohol explicitly into screening, prevention, and surveillance strategies may strengthen public health responses to substance-related harms, particularly among younger and working-age adults.

## Data Availability

The datasets analysed during the current study are publicly available through Health Canada as the Canadian Substance Use Survey (CSUS) 2023 Public Use Microdata File. Access details are provided in the CSUS PUMF User Guide.

## Abbreviations

CSUS: Canadian Substance Use Survey
PUMF: Public Use Microdata File
aOR: Adjusted odds ratio
CI: Confidence interval
CADUMS: Canadian Alcohol and Drug Use Monitoring Survey
CTADS: Canadian Tobacco, Alcohol and Drugs Survey
CADS: Canadian Alcohol and Drugs Survey
